# Conservative Management of Vulvar Cancer—Where Should We Draw the Line?

**DOI:** 10.3390/cancers16172991

**Published:** 2024-08-28

**Authors:** Neville F. Hacker, Ellen L. Barlow

**Affiliations:** 1 School of Women’s and Children’s Health, Faculty of Medicine & Health, University of New South Wales, Sydney 2052, Australia; 2 Gynaecological Cancer Research Group, School of Women’ and Children’s Health, Faculty of Medicine & Health, University of New South Wales, Sydney 2052, Australia; ellen.barlow@unsw.edu.au

**Keywords:** vulvar cancer, radical vulvectomy, radical local excision, pelvic lymph node dissection, sentinel node biopsy, surgical margins, ultrasonic groin surveillance

## Abstract

**Simple Summary:**

Vulvar cancer is a rare cancer but has a high cure rate if diagnosed early and treated appropriately. In the early 20th century, 5-year survival rates were only 15–20% because of inadequate treatment of both the primary cancer and the groin lymph nodes. By the mid-20th century, 5-year survival rates rose to 60–70% with the introduction of the radical resection of the vulva, together with the radical resection of the groin and usually pelvic lymph nodes. Although cure rates were good, physical and psychological morbidity were high with this extensive surgery. Hence, various modifications have been proposed over the past 50 years to decrease this morbidity without compromising survival, including modifications to the extent of both the vulvectomy and lymph node dissection. This paper looks at the results these modifications have on both survival and morbidity.

**Abstract:**

Vulvar cancer is a rare disease, and cure rates were low until the mid-20th century. The introduction of an en bloc radical vulvectomy and bilateral groin and pelvic lymph node dissection saw them rise from 15–20% to 60–70%. However, this very radical surgery was associated with high physical and psychological morbidity. Wounds were usually left open to granulate, and the average post-operative hospital stay was about 90 days. Many attempts have been made to decrease morbidity without compromising survival. Modifications that have proven to be successful are as follows: (i) the elimination of routine pelvic node dissection, (ii) the use of separate incisions for groin dissection, (iii) the use of unilateral groin dissection for lateral, unifocal lesions, (iv) and radical local excision with 1 cm surgical margins for unifocal lesions. Sentinel node biopsy with ultrasonic groin surveillance for patients with node-negative disease has been the most recent modification and is advocated for patients whose primary cancer is <4 cm in diameter. Controversy currently exists around the need for 1 cm surgical margins around all primary lesions and on the appropriate ultrasonic surveillance for patients with negative sentinel nodes.

## 1. Introduction

Vulvar cancer is a rare disease. The world age-standardized incidence rate is 0.85 per 100,000 women per year. The highest incidence is in Western Europe, where the rate reaches 2.4 per 100,000 women per year [1]. Squamous cell carcinomas are most common, and in younger women, vulvar squamous cell carcinoma (VSCC) is usually related to human papilloma virus (HPV) infection and has a preinvasive phase. In older women, it usually arises in areas of non-neoplastic epithelial disorders, such as lichen sclerosus [2]. The World Health Organization’s latest Classification of Female Genital Tumours, (fifth edition), simplifies the classification of a squamous lesion into HPV-associated VSCC and HPV-independent VSCC [3].

### Historical Perspectives

In the first half of the 20th century, the 5-year survival rate for vulvar cancer was 15–25% [4]. Patients were generally presented with advanced disease, and surgical procedures were poorly developed. Local excision, hemi-vulvectomy, or coagulation diathermy, sometimes combined with radiotherapy but often without lymph node dissection, were usually performed [5].

The Frenchman Basset was the first to propose a radical en bloc resection of the vulva, groin, and iliac lymph nodes, using a curved incision that extended from one anterior superior iliac spine to the other [6]. Basset performed the operation only on cadavers, but by the mid-century, Taussig in the United States [7] and Way in Great Britain [8] both utilized this approach and improved 5-year survival rates to 60–70%. After almost 40 years of experience, Way stated: “the ideal treatment of cancer of the vulva is radical vulvectomy with bilateral superficial inguinal and deep pelvic node dissection” [9]. If there was involvement of the anus or proximal urethra, some type of pelvic exenteration was combined with the radical vulvectomy.

The need for radical vulvectomy was accepted by Green, Ulfelder, and Meigs in 1958 because they believed the vulva was a “cutaneous organ”. Hence, “a truly radical vulvectomy, with wide skin margins, is all-important to assure removal not only of all the obvious malignancy but of the entire vulvar skin organ as well” [10]. In 1976, Krupp confirmed this belief, stating that the removal of the entire “field” made it available for histological study and removed possible multicentric sites [11]. The latter were reported to occur in 20–30% of cases [12].

## 2. Morbidity

Although this more aggressive approach to the primary lesion and regional lymph nodes significantly improved survival, both physical and psychological morbidity were high, so various attempts have been made to decrease morbidity without compromising survival (Table 1). 

**Figure 1:**
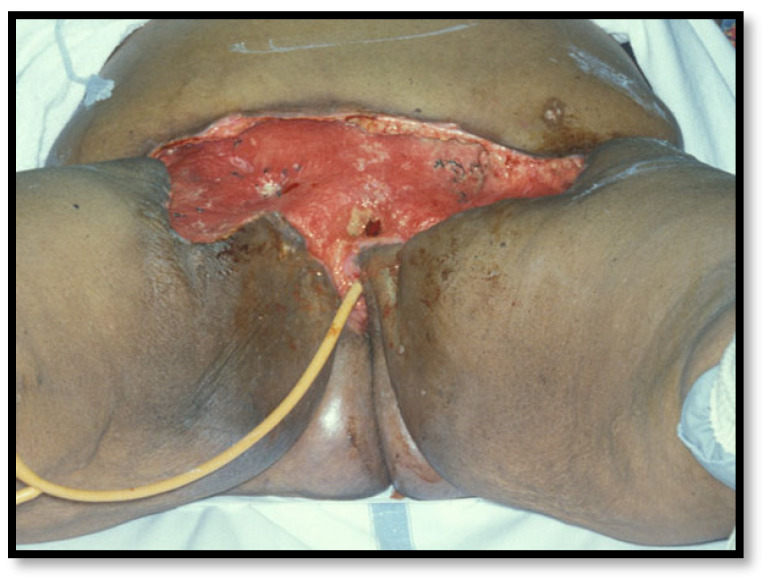
Slow granulation after an en bloc radical vulvectomy and bilateral groin dissection.

Acute morbidity was related to difficulty attaining primary wound closure, so wounds were frequently left open to granulate (Figure 1). They usually became infected, and in the early years of Way’s experience, the average post-operative hospital stay was 90 days [8].

Chronic morbidity was associated with both radical vulvectomy and lymph node dissection. The first objective evaluation of the long-term psychosexual consequences of radical vulvectomy was reported by Andersen and Hacker in 1983 [32]. They studied 15 patients who had an average interval of 5 years since vulvectomy and a mean age of 55 years. Using standard psychometric indices, they demonstrated that levels of sexual arousal were at the 8th percentile and body image at the 4th percentile compared to healthy women of comparable age.

The major long-term morbidity associated with groin lymph node dissection is lower limb lymphedema, and the reported incidence ranges from 10.9% [33] to 67% [34]. In a review of 525 groin dissections in 333 women treated for vulvar cancer at the Royal Hospital for Women in Sydney between 1987 and 2016, Barlow et al. reported an incidence of lymphedema of 35% among 392 groins undergoing complete inguino-femoral lymphadenectomy with the preservation of the fascia lata [35].

### 2.1. Measures to Decrease the Acute Morbidity of Wound Closure and Healing 

Although en bloc dissection of the groins and vulva led to a paradigmatic shift in survival for patients with vulvar cancer, problems associated with wound closure needed to be addressed.

Taussig originally followed the en bloc method described by Basset for cancer of the clitoris [7]. He stated: “In the course of the past 20 years, I found that the complications of wound healing, with wide separation of the wound edges, made it desirable to make three incisions, one over each groin and one over the vulva, retaining a bridge of normal skin between them to prevent too wide a gaping of the post-operative wound. Retention of the bridge of skin did not lead to recurrence at this point” [7]. Taussig, whose groin incision started above the inguinal ligament and extended at a 60-degree angle to the apex of the femoral triangle, did not report results specifically for this triple incision approach.

In 1962, Byron and colleagues, working at the City of Hope National Medical Center in California, also described a technique for radical vulvectomy and bilateral groin dissection using three separate incisions [36]. Their incision started at McBurney’s point, ran vertically to the inguinal ligament, then ran parallel to the ligament for about 2 cm before passing vertically downwards to the adductor–sartorius junction. The incision was used for malignant lesions of the lower extremity as well as the vulva, and in the first 83 cases, the incidence of wound breakdown was 40.9%. In 1965, Byron et al. reported the first 10 cases of vulvar cancer treated with the triple incision approach. Groin wound breakdown occurred in 50% of cases, and the mean post-operative hospital stay was 23.8 days [19].

In 1981, Hacker and colleagues reported on 100 patients from the University of California, Los Angeles (UCLA) and City of Hope National Medical Center who underwent radical vulvectomy and bilateral inguinal lymphadenectomy through separate oblique groin incisions [20]. Major wound breakdown occurred in 14% of cases, and the mean post-operative stay was 19 days. Following this paper, the separate incision approach slowly became accepted as an appropriate way to decrease morbidity without compromising survival, although it was never subjected to a randomized, prospective study. 

### 2.2. Measures to Decrease Lower Limb Lymphedema

The incidence and severity of lower limb lymphedema is proportional to the number of lymph nodes removed. In the review of the Royal Hospital for Women data, Barlow and colleagues reported an incidence of 17% when 4 or less nodes were removed, 33% when 5–8 nodes were removed, and 43% when 9 or more nodes were removed [35].

#### 2.2.1. Elimination of Pelvic Lymph Node Dissection

The initial attempt to decrease lymphedema was to eliminate the routine performance of pelvic lymphadenectomy. Green reported no positive pelvic nodes in patients with negative groin nodes in 1958 [10], and the validity of routine pelvic lymphadenectomy was questioned by Franklin and Rutledge in 1971 [13]. In 1983, Hacker et al. reviewed UCLA data and reported that all patients with positive pelvic nodes or pelvic recurrence had three or more positive unilateral groin nodes, and all had palpably suspicious groin nodes preoperatively [14]. The following year, Monaghan and Hammond, working in Stanley Way’s old unit at Gateshead, from which the policy originated, confirmed that routine pelvic lymphadenectomy was unnecessary [15].

#### 2.2.2. Modification of the Inguino-Femoral Lymphadenectomy

The first attempt to decrease lymphedema by modifying the groin node dissection occurred in 1974 when Wharton et al. defined a “microinvasive vulvar cancer” as one that was up to 20 mm in width and 5 mm in depth of invasion [16]. They suggested that groin dissection could be eliminated for such patients. Several subsequent papers demonstrated that the only patients at virtually no risk of lymph node metastases were those with lesions up to 20 mm wide and with up to 1 mm of stromal invasion [37]. FIGO subsequently classified this as stage IA. In addition, it became apparent that when groin recurrence occurred in an undissected groin, mortality was about 90% [37].

The second attempt in 1979 was to perform a “superficial inguinal lymphadenectomy”, leaving the femoral nodes intact [17]. It soon became apparent that patients were experiencing recurrence in the groin and dying after this approach [38], and this was eventually proven in a prospective study conducted by the Gynecologic Oncology Group (GOG) [39].

A third approach was to treat the groin nodes with radiation instead of surgery. The GOG conducted a prospective, randomized trial comparing inguino-femoral lymphadenectomy (and post-operative radiation if positive nodes were present) with bilateral groin and pelvic radiation [18]. This study was closed prematurely when interim monitoring revealed an excessive number of groin relapses and deaths in the radiation arm.

The first proposal to perform unilateral groin node dissection in patients with unilateral vulvar cancer and negative ipsilateral groin nodes came from Morris in 1977 [21]. Iversen proposed ipsilateral inguino-femoral lymphadenectomy for patients with unilateral stage I vulvar cancer [22], and cumulative experience with unilateral groin dissection for patients with unilateral lesions of any size 1 cm or more from the midline and negative ipsilateral nodes showed this modification to be safe.

The concept of performing nodal debulking only, followed by bilateral groin and pelvic radiation, rather than complete inguino-femoral lymphadenectomy, was first reported in 2007 [27]. The validity of this modification was confirmed by the groups in Leiden in 2015 [28] and Amsterdam in 2023 [29]. The group at Leiden also demonstrated that patients with nodal debulking had significantly less lymphedema (*p* = 0.002) [28].

#### 2.2.3. Sentinel Lymph Node Biopsy 

In 2007, van der Zee and colleagues introduced sentinel node dissection and suggested that it was safe for the treatment of cancers ≤ 4 cm in diameter [30]. As expected, short and long-term morbidity was significantly lower with this approach, and they reported a false-negative rate of only 2.5%. However, the long-term follow-up from this study reported that all 6 patients who had a false-negative sentinel node biopsy recurred in the groin and died of disease [40]. 

In addition, other investigators have usually reported false-negative rates between 5 and 10% [41,42,43], but rates as high as 27% have also been reported [44]. In a systematic review and meta-analysis of sentinel node biopsy in patients with vulvar cancer, Meads et al. reported a false-negative rate of 9% and concluded that this high false-negative rate highlighted the importance of the learning curve effect, which will always be a problem with rare cancer [45].

We have never believed that sentinel node biopsy should be regarded as the standard of care for patients with vulvar cancer because most patients, properly informed, are not prepared to take a small risk of death from vulvar recurrence in exchange for the opportunity to avoid lymphedema. In a patient preference study in 2014, Farrell et al. gave 60 women who had been treated for vulvar cancer a choice between a 60% risk of lymphedema but virtually no risk of death from groin recurrence if they had a full groin dissection versus a 1% risk of death from groin recurrence but minimal risk of lymphedema if they had a sentinel node biopsy [46]. Fifty-three percent of patients said they would take no risk at all with their lives, and only 15% said they would be prepared to take the risk required (1 in 100).

In 2018, the Dutch group followed 79 patients with negative sentinel nodes with a serial groin ultrasound every 3 months for 2 years. Two false negatives were discovered, but both patients were long-term survivors after groin dissection and adjuvant radiation [31]. We now believe that sentinel node biopsy and serial ultrasonic surveillance of the groin(s) in patients with a negative sentinel node should be the standard of care for patients with squamous cell carcinomas of the vulva 4 cm or less in diameter. Based on a pilot study of ultrasonic surveillance, which only we reported from the Royal Hospital for Women in Sydney in 2023, we believe that groin ultrasound should occur every 2 months and be continued for at least 12 months [47].

### 2.3. Measures to Decrease Psychosexual Morbidity by Vulvar Conservation

The distressing statistics regarding the psychosexual consequences of radical vulvectomy [32] led to more conservative effects with vulvar resection. DiSaia et al. were the first to suggest a more conservative vulvar resection in 1979 [17]. They suggested a “wide local excision” for a lesion 2 cm or less in diameter with up to 5 mm of stromal invasion and suggested surgical margins of 3 cm.

In 1981, Iversen et al. proposed hemi-vulvectomy for unilateral lesions 2 cm or less in diameter with stromal invasion of any depth, although they only performed hemi-vulvectomy in 2 out of 117 cases (1.7%) [22]. They recommended radical vulvectomy for all midline lesions.

In 1984, Hacker et al. reported a retrospective study of 84 patients with stage one vulvar cancer (2 cm or less in diameter with negative nodes) from UCLA [23]. Fifty-six patients had a radical vulvectomy, and 28 had what they called a “radical local excision”, implying both a wide and a deep resection of primary cancer. The incidence of local invasive vulvar recurrence was 4% in both groups.

In a review of 135 patients from UCLA and City of Hope National Medical Centers in 1990, Heaps et al. reported that there were no vulvar recurrences if the histological margin following a radical vulvectomy or radical local excision was at least 8 mm [24]. Assuming a 20% tissue shrinkage with formalin fixation, this translated to a surgical margin of at least 1 cm. This guideline for conservative vulvar surgery was subsequently reproduced in other studies [48,49] and was widely accepted for the next 20 years.

**Figure 2:**
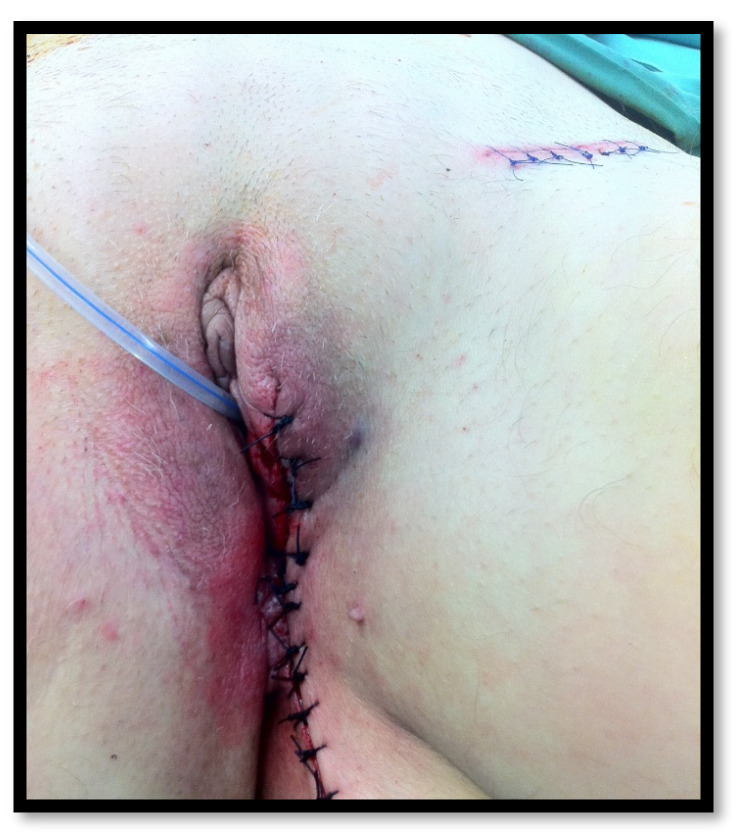
Radical local excision for a left postero-lateral lesion and unilateral inguino-femoral lymphadenectomy.

Conservative vulvar resection was subsequently extended to include unifocal cancers of any size that were confined to the vulva (Figure 2). In 2007, DeSimone et al. reported the results of 61 patients with a lateral T1 squamous cell vulvar carcinoma and 61 patients with a lateral T2 lesion who were treated at the University of Kentucky from 1963 to 2003 [50]. Radical vulvectomy was performed in 60 patients (49.2%) and radical hemi-vulvectomy in 62 (50.8%). The aim was to have surgical margins of at least 1 cm in both groups. Local and distant recurrences were the same in each group, and 96.7% of patients were alive and free of disease at 5 years.

In 2008, Tantipalakorn et al. reviewed our results with the same group of patients (cancers confined in the vulva with no clinically suspicious groin nodes) treated at the Royal Hospital for Women in Sydney from 1987 through 2005 [25]. There were 121 such patients, and 116 (95.9%) were treated with radical local excision. Only 5 patients (4.1%) underwent radical vulvectomy because of tumor multifocality. We aimed to obtain a 1 cm surgical margin in all patients. With a median follow-up of 84 months, the overall survival at 5 years was 96.4%. Local recurrences occurred in 21.5% of cases, but 96% of these were cured by surgical re-excision or radiation therapy. 

With larger lesions, various plastic reconstructive techniques may help to decrease the morbidity associated with the closure of the vulvar wound. Giannini et al. reported that the use of the V-Y gluteal fold advancement flap correlated with an increased rate of adequate surgical margins and a reduced need for adjuvant radiotherapy [26].

## 3. Types of Local Recurrence

In 2002, Rouzier et al. from Paris reviewed the records of 215 consecutive patients that they treated in a single institution from 1978 to 1999 [51]. For the first time, they described two types of vulvar recurrence — “primary site”, which occurred within 2 cm of the vulvectomy scar, and “remote site”, which occurred more than 2 cm from the vulvectomy scar. They also recorded skin bridge recurrences, which had been of concern since the introduction of separate groin incisions [20].

A surgical margin less than 1 cm was an independent risk factor for primary site recurrence (*p* < 0.002), and the mean interval from surgery was 13 months. The mean time to remote site recurrence was 33 months, and the only independent risk factor was the presence of an adjacent dermatosis (*p* < 0.001) [51]. Rouzier hypothesized that remote recurrences may be new primary lesions arising in vulvar epithelial disorders, as opposed to true recurrences. Stanley Way made the same suggestion in 1966 [52].

### 3.1. Controversies Regarding Surgical Margins

Since 2010, several authors have questioned the need for surgical margins of 1 cm [53,54,55,56,57,58,59,60]. These were all based on relatively small retrospective studies, and none classified the recurrence as being at the primary or remote site.

In 2016, Nooij et al. from Leiden University reported a meta-analysis of 1278 patients from 10 studies and a cohort study of 148 patients from their own institution. While the meta-analysis demonstrated that a tumor-free space of < 8 mm was associated with a higher risk of local recurrence, a tumor-positive margin was the only independent risk factor for local recurrence in their small cohort study [58].

In 2018, te Grootenhuis et al. conducted a systematic review of prognostic factors for the local recurrence of squamous cell carcinoma of the vulva [59]. Data from 3657 patients and 22 studies were included in the review. They concluded that the current quality of data did not allow for evidence-based clinical decision making but then said that based on their current review, “there seems to be no lower limit (apart from involved margins) below which treatment (either re-excision or adjuvant radiotherapy) to the vulva should be considered”. 

Regarding the division into primary and remote site local recurrences, they considered this to be “too arbitrary, not reproducible, and should be abandoned” [59]. The following year, in a study of 287 patients from two Dutch centers, the same group reported that local recurrences in patients with primary vulvar carcinoma were associated with dVIN (with or without lichen sclerosus) in the pathological margin rather than any tumor-free margin distance [60]. 

Also, in 2018, Micheletti et al. reported on the prognostic impact of reduced tumor-free margin distance on the long-term survival of patients with FIGO stage IB/II vulvar squamous cell carcinoma [61]. This paper is the first to study node-negative patients only, but there were only 114 eligible patients. Although no differences were found in the local recurrence rate, overall and disease-specific survival was significantly worse in patients with margins < 5 mm. They recommended that these patients should be offered further surgical or adjuvant treatment. 

In 2020, we reviewed our own 30-year experience with vulvar cancer at the Royal Hospital for Women in Sydney, looking specifically at the significance of surgical margins [62]. The sites of all primary cancers were accurately recorded prospectively, and all unifocal vulvar cancers were treated by radical local excision, with the aim of achieving a surgical margin of 1 cm of unstretched skin. All vulvar intraepithelial neoplasia (VIN) was superficially excised with clear but close margins. Data were obtained on 345 consecutive patients, with a median follow-up of 93 months. The 5-year disease-specific survival was 86%.

There were 78 vulvar recurrences (22.6%), of which 33 (42.3%) were at the primary site and 45 (57.7%) at a remote site. Interestingly, the majority of recurrences were at a remote site, which may explain why smaller studies are unable to show any significance for the margin width. 

The median interval from initial treatment to primary site recurrence was 20 months, while it was 39 months for remote site recurrence. In multivariate analysis, a margin < 5 mm was associated with a higher risk of all vulvar and primary site recurrences, while a margin 5 to <8 mm was associated with a higher risk of primary site recurrences [62].

There were 27 vulvar recurrences in patients with a histological margin < 8 mm, of which 26 (96.3%) were at the primary site and one at a remote site (*p* < 0.001). Of the 51 recurrences in patients with margins ≥ 8 mm, 44 (86.3%) were at a remote site and seven were at the primary site (*p* < 0.001). For patients with margins of <5 mm, and treatment with either radiotherapy or re-excision, decreased the rate of primary site recurrence from 40 to 4.3% (*p* = 0.003). Treatment for margins 5 to 7.9 mm also reduced the risk of recurrence from 25.5 to 11.1%, but this was not statistically significant [62].

In our opinion, these data strongly reaffirm Rouzier’s subdivision of local recurrences into primary and remote site lesions [51], with the remote site lesions considered to be new primary cancers. They also confirm the need to maintain a surgical margin of 1 cm of unstretched skin and to treat close margins by surgical re-excision or radiation, particularly if the margin is less than 5 mm. 

Also, in 2020, Yang et al. reported on tumor-free margins and local recurrence in squamous cell vulvar carcinomas from the Mayo Clinic. Data were obtained on 335 patients from the three sites of the clinic [63]. They concluded that patients with tumor-free margins < 8 mm had a higher local recurrence rate. The Mayo group did not break local recurrences into primary and remote site recurrences, but the large number of patients in the study allowed them to achieve statistical significance for vulvar recurrence with a margin < 8 mm.

### 3.2. Field Cancerization 

The concept of “field cancerization” was introduced by Slaughter et al. in 1953 following a study of 783 patients with oral cancer [64]. It is now recognized that genetically altered but histologically normal-appearing cells may be present adjacent to occult cancers. With technological advances in the future, it should be possible to identify such cells and tailor surgical margins. This will be particularly helpful when the primary lesion is close to the clitoris, urethra, or anus [65]. 

## 4. Conclusions

After 40 years of experience, Stanley Way concluded in 1978 that the optimal treatment of vulvar cancer should be en bloc radical vulvectomy, bilateral inguino-femoral lymphadenectomy, and bilateral pelvic lymphadenectomy [8]. This approach gave optimal results in terms of survival but was associated with major physical and psychosexual morbidity. Many modifications have been proposed in the last 50 years to try to improve morbidity without compromising survival. To answer the question posed in the title, the methods that have achieved this goal have been the elimination of routine pelvic lymphadenectomy, the use of separate groin incisions, unilateral groin dissection for unifocal, unilateral lesions, radical local excision with a surgical margin of 1 cm of unstretched skin for unifocal lesions, and sentinel node biopsy and serial ultrasonic surveillance of the groin(s) for patients with unifocal lesions 4 cm or less in diameter if they have node-negative disease.

The recent VULCAN study [66] confirmed earlier European studies [67,68] that the case volume per treating institution is an important prognostic factor. 

## 5. Future Directions

Although early results suggest that ultrasonic groin surveillance can detect patients with false-negative sentinel nodes at a time when most can be cured, more research is needed to determine the most appropriate interval between scans (e.g., 6, 8, or 12 weeks) and the most appropriate duration of ultrasonic surveillance (e.g., 12, 18, or 24 months). Future research is also needed to investigate the genetic profile of histologically normal skin around the primary lesion, particularly if the lesion is close to the clitoris, urethra, or anus. This may allow much closer margins if the genetic profile is normal or suggest the need for primary or adjuvant radiation if the profile is abnormal.

## Figures and Tables

**Table 1:** Modifications to en bloc radical vulvectomy and bilateral groin and pelvic lymphadenectomy, and their effects on survival.

Modification	Effect on Survival
Elimination of pelvic lymphadenectomy [10,13,14,15,16]	Nil
No LND for cancer ≤ 2 cm diameter with ≤5 mm invasion [16]	Decreased
“Superficial” groin dissection [17]	Decreased
Radiation instead of groin dissection [18]	Decreased
Use of separate groin incisions [7,19,20]	Nil
Unilateral groin LND for lateral lesions [21,22]	Nil
RLE for lesions ≤ 2 cm diameter [17,22,23]	Nil
I cm surgical margins for unifocal cancers [24]	Nil
RLE for all unifocal lesions [25,26]	Nil
Nodal debulking instead of full groin LND [27,28,29]	Nil
Selective sentinel node biopsy [30]	Small decrease
Selective SNB + ultrasonic surveillance of sentinel node negative groins [31]	Nil

LND: lymph node dissection; RLE: radical local excision.

## Data Availability

All data are available within the paper and cited references.
